# Resection of rectal metastasis after previous radical surgery for pancreatic cancer: Case report and literature review

**DOI:** 10.1097/MD.0000000000036365

**Published:** 2023-12-08

**Authors:** Shuwei Chen, Yanfei Hao, Shaoyang Huang, Dong Leng, Yuxiang Ma

**Affiliations:** a Department of Gastrointestinal Surgery, Chinese PLA 970th Hospital, Yantai, P.R. China; b Department of Anesthesiology, Yantai Hospital of Traditional Chinese Medicine Hospital, Yantai, P.R. China; c Information Department, Chinese PLA 970th Hospital, Yantai, P.R. China.

**Keywords:** ductal adenocarcinoma, pancreatic cancer, rectal metastasis, surgical resection

## Abstract

**Rationale:**

Pancreatic ductal adenocarcinoma (PDAC) is the main type of pancreatic cancer with a poor prognosis. Rectal metastasis after radical resection of PDAC is comparatively rare, and the understanding of such cases is currently not unified. This study presents the entire process of diagnosis and treatment of a patient with PDAC metastasized to the rectal. We propose the viewpoint of exploring potential biomarkers or establishing effective predictive models to assist in the clinical decision-making of such cases.

**Patient concerns:**

We present the case of a 71-year-old man with slight abdominal distension and dull pain. He underwent surgical treatment for a malignant tumor of the pancreatic body, which was discovered through computed tomography and magnetic resonance imaging examinations. Nine months after the pancreatectomy, a rectal mass was identified by digital rectal examination and diagnosed as a malignant lesion through a puncture biopsy. After a multidisciplinary joint consultation, the patient underwent radical surgery. It was later confirmed as rectal adenocarcinoma based on postoperative pathological results.

**Diagnosis:**

The pathological result after pancreatic surgery was PDAC, which had invaded the peripheral nerves and abdominal arteries. A diagnosis of rectal metastasis was determined ultimately by combining with the medical history and immunohistochemical staining results.

**Interventions and outcomes:**

Treatment of the PDAC included laparoscopic resection of the body and tail of the pancreas combined with splenectomy, and postoperative systemic chemotherapy. In addition, treatment of the rectal metastasis included laparoscopic radical resection and postoperative systemic chemotherapy. The patient’s current living condition was good.

**Lessons:**

As a rare metastatic site of PDAC, rectal metastasis should be avoided because of misdiagnosis and missed diagnosis. Surgical resection is still an effective treatment strategy for localized pancreatic tumors and isolated metastases. Furthermore, the mining of potential biomarkers or the establishment of predictive models for pancreatic cancer and its metastases may contribute to better clinical decision-making in the future.

## 1. Introduction

Pancreatic cancer is a common malignant tumor of the digestive tract, and its global incidence and mortality were ranked 12th and 7th, respectively in 2020.^[1]^ The prognosis of patients with pancreatic cancer is usually poor, with an overall 5-year survival rate of approximately 5%.^[2]^ Pancreatic ductal adenocarcinoma (PDAC) is the main type of pancreatic cancer, accounting for 80% to 90%.^[3,4]^ Due to its insidious onset and rapid progression, most patients are already in locally advanced stages (such as vascular or neural invasion)^[5,6]^ or metastases (such as liver metastasis and peritoneal transmission)^[7,8]^ at diagnosis.

Although the 5-year survival rate of patients undergoing surgical resection is not high (approximately 10% to 25%), surgery is still the only treatment with the potential to cure PDAC in terms of resectability status.^[9]^ Most patients with pancreatic cancer are at a high risk of postoperative recurrence and metastasis, including recurrence at the surgical site, abdominal lymph node metastasis, and distant metastasis (such as liver and lung).^[10,11]^ In contrast, rectal metastases are atypical and extremely rare. However, there is no consensus regarding the management of recurrent pancreatic cancer after surgery.^[12,13]^

Here, we report the case of a patient with unusual metachronal metastases to the rectum who underwent initial curative pancreatectomy approximately 9 months ago. Surgical resection of the rectal metastasis and postoperative chemotherapy were selected with satisfactory results.

## 2. Case presentation

**Figure 1.**
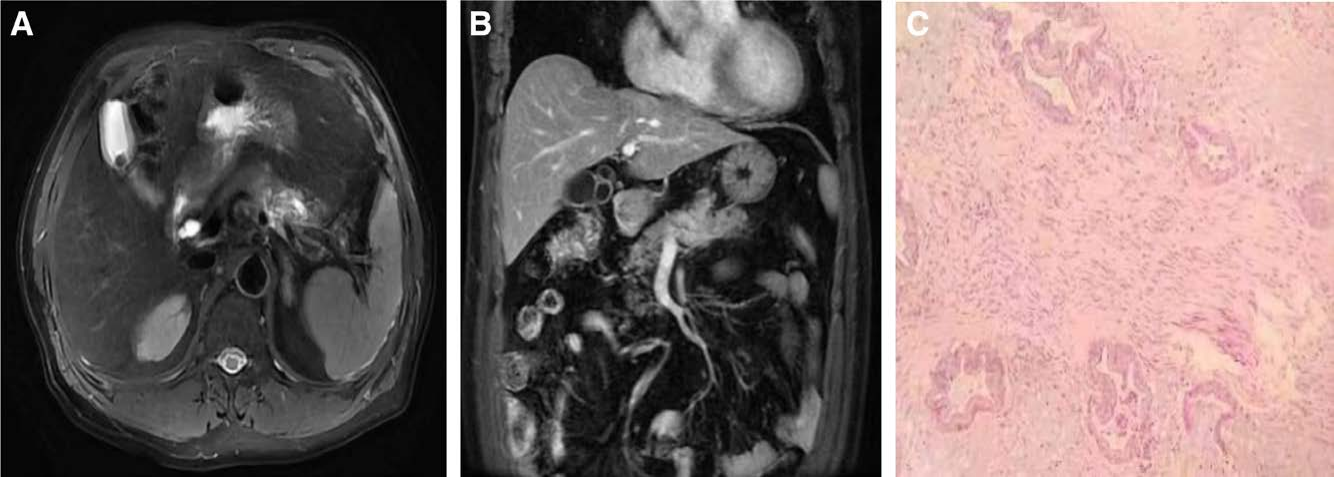
Primary pancreatic tumor. Contrast-enhanced CT scans (A. The abdomen in transverse view, B. coronal plane of the abdomen) showed that a pancreatic mass was mainly located in the body and tail of the pancreas, adjacent to the left gastric artery and spleen artery. (C) Pathology confirmed the pancreatic ductal adenocarcinoma (PDAC). CT = computed tomography.

A 71-year-old man was admitted with slight abdominal distension and dull pain in the left side of the abdomen. Blood tests showed a significant increase in carbohydrate antigen 19-9 (97.19 U/mL) while abdominal ultrasound indicated a decrease in the density of the pancreatic body and tail. He further underwent computed tomography and magnetic resonance imaging, which revealed a pancreatic body tumor with invasion of the left gastric artery, spleen artery, coronary vein, and transverse mesocolon (Fig. 1). On the basis of intraoperative exploration, the patient underwent laparoscopic resection of the body and tail of the pancreas combined with splenectomy on March 17, 2022. The postoperative pathological result was highly to moderately differentiated PDAC, with a size of approximately 5 × 3 cm (Fig. 1), which was consistent with R0 resection. Nerve and abdominal artery invasions were observed, and the peripheral lymph nodes showed reactive hyperplasia. Finally, we diagnosed it as T4N0M0 according to the eighth edition of the American Joint Committee on Cancer (AJCC) TNM staging system.^[14,15]^ Immunohistochemical analysis showed that the positive expression rate of Ki-67 protein was 10% and was negative for c-erbB-2, epidermal growth factor receptor, and postmeiotic segregation increased 2. From April 26, 2022, 6 courses of chemotherapy with gemcitabine (intravenous infusion of 1800 mg, days 1 and 8, 3 weeks per cycle) and capecitabine (1.5 g, days 1 to 14, 3 weeks per cycle) were administered. Pre-chemotherapy blood tests revealed tumor markers with elevated carbohydrate antigen 72-4 levels (16.03 U/mL). During chemotherapy, the patient was diagnosed with type 2 diabetes, and his blood sugar level was well controlled after insulin treatment.

**Figure 2.**
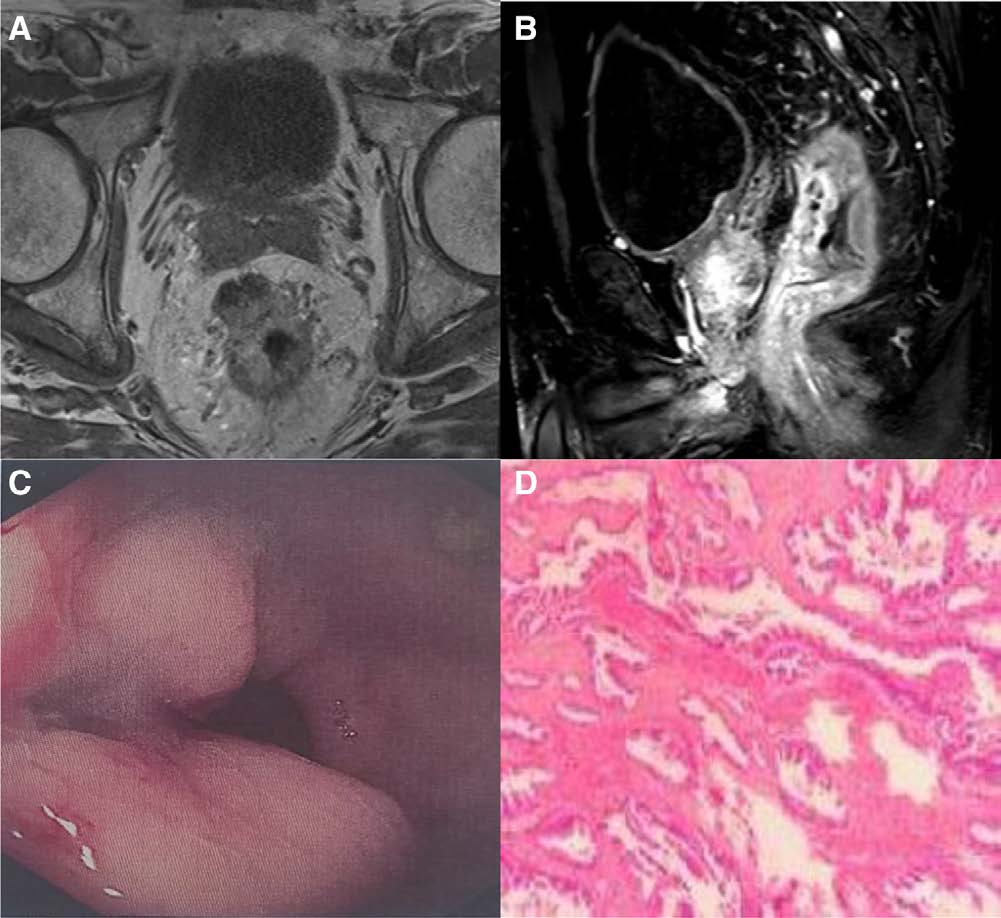
Secondary rectal tumor. Enhanced MRI scans (A. The abdomen in transverse view, B. sagittal plane of the abdomen) showed cystic and solid lesions in the anterior wall of the rectum. (C) A protruding mass under the rectal mucosa was revealed by colonoscopy. (D) Histology indicated an adenocarcinoma, and the first consideration was metastasis or infiltration of PDAC. MRI = magnetic resonance imaging, PDAC = pancreatic ductal adenocarcinoma.

Nine months after the pancreatectomy, the patient visited the outpatient clinic for digital rectal examination due to constipation and a rectal lump located on the anterior wall of the rectum 4 cm away from the anus was found. After admission, further enhanced magnetic resonance imaging revealed cystic and solid lesions in the anterior wall of the rectum, indicating a tumor-related lesion (Fig. 2), while colonoscopy revealed a submucosal mass in the rectum (Fig. 2). To further clarify the diagnosis, a puncture biopsy was performed, and the pathological results indicated pancreatic ductal adenocarcinoma infiltration. To understand the patient’s overall condition, further whole-body positron emission tomography-computed tomography examination revealed cystic thickening of the intestinal wall in the upper and middle segments of the rectum, which was considered a metastatic lesion, and no other potential metastases were found.

After a multidisciplinary joint consultation, the patient underwent laparoscopic radical resection of the rectal cancer on October 24, 2022. Intraoperative exploration revealed that the tumor was located within the rectal bladder lacuna and had infiltrated the rectum. The postoperative pathological result was rectal adenocarcinoma (Fig. 2), and no tumor infiltration or metastasis was observed after cleaning the surrounding lymph nodes. Subsequent immunohistochemical staining results showed that cytokeratin 7, CDX2 protein, Ki-67 protein, mucoprotein 1, and mucoprotein 6 were all positive, whereas cytokeratin 20 and mucoprotein 2 were negative. Combined with the medical history and pathological results of the original pancreatic cancer, a conclusion of rectal metastasis was drawn. From January 20, 2023, the patient was treated with 6 courses of paclitaxel injection (albumin-bound) (200 mg, days 1 and 8, 3 weeks for 1 cycle), TS-1 (40 mg, twice a day, 3 weeks per cycle), and nimotuzumab injection (200 mg, once a week, 3 weeks per cycle). The full details of the diagnosis and treatment are shown in Table 1.

**Table 1:** The timeline of the treatment process in the present case.

Time	Preoperative diagnosis	Surgery/invasive operation	Postoperative treatment
October 2021	Abdominal distension and dull pain		
March 08, 2022	Elevated serum CA19-9		
March 13, 2022	Pancreatic tumor (CT and MRI)		
March 17, 2022		Laparoscopic pancreatectomy combined with splenectomy	
April 26, 2022	Elevated serum CA72-4		
April 26, 2022			Chemotherapy (gemcitabine and capecitabine)^[1]^
May 17, 2022			Chemotherapy (gemcitabine and capecitabine)^[2]^
May 18, 2022	Type 2 diabetes		
June 07, 2022			Chemotherapy (gemcitabine and capecitabine)^[3]^
June 28, 2022			Chemotherapy (gemcitabine and capecitabine)^[4]^
July 19, 2022			Chemotherapy (gemcitabine and capecitabine)^[5]^
August 09, 2022			Chemotherapy (gemcitabine and capecitabine)^[6]^
October 10, 2022	Constipation		
October 12, 2022	A colonoscopy revealed a rectal mass		
October 17, 2022	Biopsy showed PDAC infiltration		
October 21, 2022	Rectal metastasis (PET-CT)		
October 24, 2022		Laparoscopic radical resection of rectal cancer	
October 27, 2022	CT examination revealed ascites		
October 28, 2022		Percutaneous puncture and drainage of abdominal fluid	
January 20, 2023			Chemotherapy with paclitaxel for injection (albumin-bound), TS-1 and nimotuzumab injection (3 weeks for 1 cycle, 6 cycles)

CA19-9 = carbohydrate antigen, CA72-4 = carbohydrate antigen 72-4, CT = computed tomography, MRI = magnetic resonance imaging, PET-CT = positron emission tomography-computed tomography.

**Figure 3.**
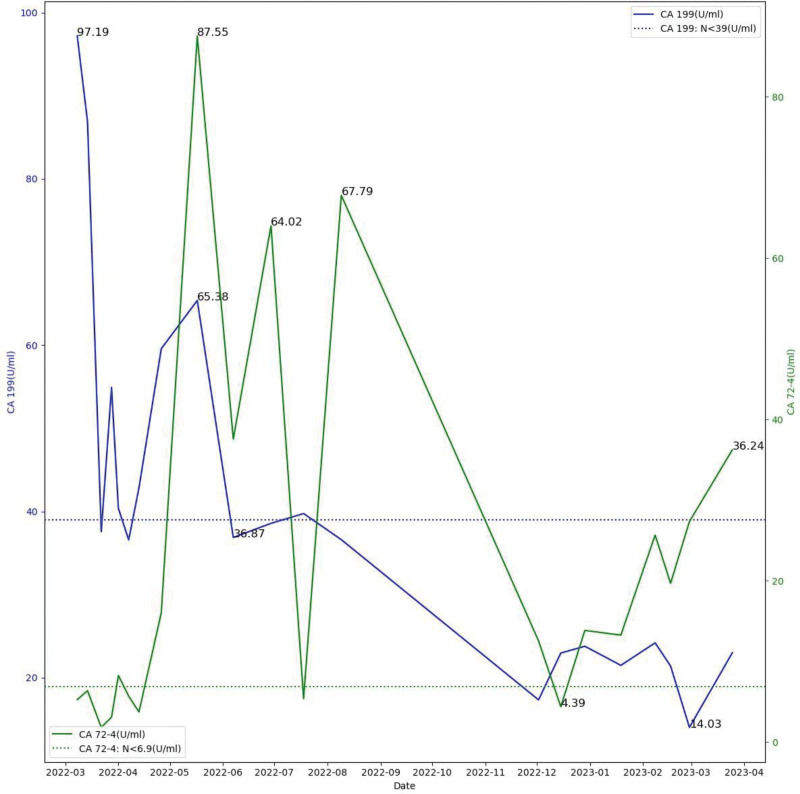
The change curve of serum tumor markers. CA19-9 = carbohydrate antigen 19-9, CA72-4 = carbohydrate antigen 72-4.

No severe postoperative complications resulted from either surgery, and no serious side effects were caused during any chemotherapy period. The patient’s current living condition was good, and there were no obvious abnormalities on imaging examination. The patient was reviewed for changes in tumor markers during routine follow-up (Fig. 3). Informed consent was obtained from the patient.

## 3. Discussion

PDAC, a malignant tumor of the digestive tract with high mortality, has atypical early symptoms and is prone to metastasis. Most patients have already experienced distant metastasis at the time of diagnosis, thus losing the opportunity for radical surgery.^[16]^ The specific tumor location determines the specific surgical procedure, including pancreaticoduodenectomy and distal or total pancreatectomy. For possible resection of pancreatic cancer, neoadjuvant treatment can be performed first to achieve R0 resection, which is significantly longer than the survival period of R1 resection.^[17]^ Postoperative chemotherapy has been shown to be effective in improving patient survival, especially for small tumors.^[18–20]^ Although medical and surgical techniques for pancreatic cancer treatment have made great progress in the past few decades, they still face enormous challenges. Given the occult and highly invasive nature of pancreatic cancer, early diagnosis and treatment have always been key to improving the overall prognosis of PDAC.

In general, the main mechanisms of pancreatic cancer metastasis include lymphatic metastasis, hematogenous metastasis, peritoneal implantation, and direct invasion. Based on the frequency of metastatic sites, they can be divided into common, relatively common, uncommon, and rare. However, the rectum is a rare site of metastases from pancreatic cancer, as only a few cases have been reported with different outcomes.^[21–23]^ Sun J et al reported a case of rectal metastasis from pancreatic carcinoma 2 years after pancreatoduodenectomy, which resulted in an increased tumor load after radiotherapy and chemotherapy.^[24]^ Another case of rectal metastasis of pancreatic acinar cell carcinoma 28 months after curative pancreatectomy was reported by Ohara Y et al, who treated the patient with abdominoperineal resection. Metastasis of rectal cancer to the pancreas has also been reported^[25]^; therefore, blood and lymph node metastasis may be the main routes for tumor metastasis between the pancreas and rectum. Anatomically, tumor cells can break through the pancreatic capsule and directly seed the rectal serosa to form retroperitoneal implantation metastases, which is also attributed to the fact that both the pancreas and rectum are extraperitoneal organs. Functionally, as an exocrine malignant tumor, detached cancer cells of PDAC have the opportunity to enter the intestinal tract with pancreatic juice secretion and grow into metastatic cancer owing to their prolonged rectal mucosa. In this case, the surrounding nerves and blood vessels have been invaded by the tumor and there is a high possibility of blood or implantation metastasis due to tumor cell detachment.

Rectal metastases are rare and usually occur in patients with advanced tumors, including those with synchronous or metachronous metastatic disease. Metastatic tumors mainly originate from the stomach, breast, and ovaries, and are often accompanied by symptoms such as changes in bowel habits, bloody stools, and constipation. Based on the above information, the treatment options for rectal metastases are particularly crucial and require rigorous evaluation. After strict examination to eliminate the possibility of metastasis to other sites, surgical resection is the best treatment option. In addition, chemotherapy based on pathological results and radiotherapy is currently an effective method for reducing the tumor burden in patients. In summary, the early diagnosis of rectal metastatic cancer is of great significance for determining the treatment and prognosis of patients, and survival time can be prolonged by effective treatment before widespread metastasis. In this case, the preoperative biopsy result was considered to be rectal adenocarcinoma infiltrated by pancreatic ductal adenocarcinoma, and positron emission tomography-computed tomography examination showed no metastasis to other sites, so the patient underwent radical resection surgery as soon as possible.

Many risk factors for the occurrence and development of pancreatic cancer have been summarized, but there are no satisfactory specific markers for screening and monitoring.^[2]^ The most commonly used clinical marker is carbohydrate antigen 19-9,^[26]^ and potential effective biomarkers are still under continuous investigation. Even patients who have undergone surgical treatment would require long-term comprehensive follow-up, and few metastatic tumors can be detected and treated with surgical resection, as in this case. Owing to the high invasiveness and heterogeneity of pancreatic tumors, there is currently no unified and effective chemoradiotherapy regimen for most patients without the opportunity for surgery. However, discovering new tumor biomarkers with guiding significance for diagnosis and treatment may require extensive genomics and proteomics experiments in the future.

With the continuous integration of medicine and engineering, based on the comprehensive information of patients, some models can be established to assist in clinical decision-making, including diagnostic prediction models (identification of high-risk groups of pancreatic cancer), treatment prediction models (treatment selection based on blood tests, imaging, and pathological findings), and prognosis prediction models (prediction of postoperative recurrence and metastasis). Although the implementation and application of the above models can alleviate the physical and mental stress and economic burden of patients to a certain extent, their development and validation need to be based on a large number of cases collected through further retrospective or prospective studies.

## 4. Conclusion

Metachronous rectal metastases from pancreatic body ductal adenocarcinoma are rare. Our case focuses on the diagnosis and treatment of a patient with rectal metastasis from PDCA, who currently has a good living condition. The rectum, as a rare metastatic site of PDCA, must be paid great attention to avoid misdiagnosis and missed diagnosis. Surgical resection and postoperative systemic chemotherapy are effective treatment strategies for pancreatic tumors and isolated metastases. The exploration of potentially effective biomarkers and the development of stable prediction models could play a positive role in clinical decision-making for pancreatic cancer in the future.

## Acknowledgments

We thank all medical staff and the patient who participated in this study.

## Author contributions

**Conceptualization:** Shuwei Chen, Yanfei Hao, Yuxiang Ma.

**Investigation:** Shuwei Chen, Yanfei Hao, Shaoyang Huang, Dong Leng.

**Resources:** Shuwei Chen, Yanfei Hao, Shaoyang Huang, Dong Leng, Yuxiang Ma.

**Writing – original draft:** Shuwei Chen, Yanfei Hao, Shaoyang Huang, Dong Leng.

**Writing – review & editing:** Yuxiang Ma.
